# The Design and Manufacture of a Multilayer Low-Temperature Protective Composite Fabric Based on Active Heating Materials and Passive Insulating Materials

**DOI:** 10.3390/polym11101616

**Published:** 2019-10-05

**Authors:** Yanli Sun, Rui Wang, Bo Li, Wei Fan

**Affiliations:** 1 School of Textile Science and Engineering, Xi’an Polytechnic University, Xi’an 710048, China; sunyanli@xpu.edu.cn (Y.S.); 1988li01bo22@163.com (B.L.); fanwei@xpu.edu.cn (W.F.); 2 Key Laboratory of Functional Textile Material and Product, Ministry of Education, Xi’an Polytechnic University, Xi’an 710048, China; 3 School of Textiles Science and Engineering, Tiangong University, Tianjin 300387, China

**Keywords:** low-temperature protective, the composite fabric, phase change microcapsules, SiO_2_ aerogel

## Abstract

Based on active heating materials (the phase change microcapsules (microPCMs)) and passive insulating materials (SiO_2_ aerogel), a new-type multilayer low temperature protective composite fabric (MPF) was designed and manufactured to meet the demands of protection and operation in a short time under a low-temperature environment. Results showed that the MPF consisted of three layers including the fabric layer, the microPCM function layer, and the SiO_2_ aerogel thermal insulation layer. The differential scanning calorimeter (DSC) results demonstrated that the phase transition enthalpy of the composite was 96.2 J/g during the cooling process. The low-temperature resistance and thermal insulation performance at −50 °C were investigated. The results also demonstrated that the low-temperature resistance time of the MPF was 660 s and the power consumption of the MPFs needed to maintain 37 °C for 10 and 20 min were 629 J and 1872 J, respectively. Compared with the microPCM function layer and the thermal insulation layer, which have the same thickness as the MPF, the low-temperature resistance time of the MPF was prolonged for about 2 and 3 min, respectively. The MPF could provide effective protection of the low-temperature work in a short time and could be applied as potential materials in low-temperature protection.

## 1. Introduction

With the development of science and technology, the environmental conditions of human contact have become more complicated. It is sometimes necessary to work in low-temperature environments, such as in low-temperature tests in the field of biomedicine, gun training for border guards in cold regions, and outdoor operation equipment for polar work. Although the time of some operation processes is short, it is still essential to wear protective products to avoid harm to the operator caused by low temperatures [1,2]. The prerequisite for the preparation of protective products is to choose proper thermoregulating materials. At present, thermoregulating materials can be divided into passive insulating materials and active heating materials in the form of heat source control [3,4].

Passive insulating materials can prevent the rate of heat loss by reducing heat conduction, convection, and radiation in the human body, materials, and the environment. To suppress the body heat dissipation into the environment, the porous structure of the materials can be used as passive insulating materials. A low thermal convection and conduction was obtained because the porous structure contains a significant amount of air [5]. Conventional textile materials, such as cotton, wool, feather, ultrafine fiber, and hollow fiber, are commonly used insulating materials. However, these materials mainly achieve their excellent thermal insulation properties by increasing their density and thickness, which will make the protective product heavy and thus limit the flexibility of human activities. Aerogel is well known as a nano-porous solid material with the lightest weight, high surface area, high porosity, and low thermal conductivity [6,7,8]. These properties suggest a broad application for aerogels in many fields, especially for thermal insulation. The SiO_2_ aerogel has been widely studied, and its preparation technology is mature. The unique structure of the SiO_2_ aerogel, the nanometer network, and the three-dimensional nano-porous structure significantly restrict gaseous thermal conduction, and its thermal conductivity is very low. However, the structure of the SiO_2_ aerogel results in poor mechanical properties, which must be solved via the introduction of reinforcing phases. Fibers or fabrics are often selected for the reinforcing phase and form an SiO_2_ aerogel composite with excellent thermal insulation properties. Fei et al. [9] compounded a glass fiber/polyimide/SiO_2_ composite aerogel by dispersing glass fibers in a polyimide/SiO_2_ hybrid solution. The composite aerogel had low thermal conductivity (0.0268–0.028 W·m^−1^·K^−1^). Wang et al. [10] used an SiO_2_ aerogel to prepare the thermal insulation composite. Waterborne polyurethane was mixed with modified silica aerogel and then coated on the surface of the fabric. Compared with the original fabric, the thermal insulation rate of the composite fabric was improved twofold.

Active heating materials can actively provide heat to the human body by implanting heating devices or using heating fibers/materials, such as hygroscopic thermal fibers, electrical heating fabrics, chemical heat storage materials, solar thermal storage materials, and phase change materials (PCMs). Hygroscopic heating fibers, such as Warmsensor fibers (Toray, Tokyo, Janpan), EKS fibers (Toyobo, Tokyo, Janpan), etc., can absorb water vapor from the external environment or human body and generate heat because it contains a large number of hydrophilic groups [11]. Electrical heating fabric is made by embedding heating elements into the fabric or the clothing, and allowing the the human body to gain heat by using the power source to convert the electric energy into heat energy [12,13]. Chemical heat storage materials can provide a certain amount of heat to the human body through some chemical reactions. For example, iron powder/tourmaline is mixed into the spinning solution during the process of polymer spinning, and iron powder/tourmaline reacts with oxygen in the air/water on the surface of human body to produce heat [14,15]. A solar thermal storage material, such as Solar-α, Thermotron fiber (Unitika, Tokyo, Janpan), Thermocatch fiber (Mitsubishi Rayon, Tokyo, Janpan), etc., absorbs the visible and near-infrared rays of solar radiation, and then reflects the human body’s heat radiation to achieve a thermal insulation effect [16]. Incorporating PCMs into fiber or fabric provides thermoregulating function to textiles [17,18]. PCMs can absorb or release heat energy as its own phase changes at a defined range of temperatures and can be used repeatedly to reduce heat energy waste [19,20]. The developments of active heating materials offer the possibility to prepare light-weight and low-temperature protection products. While there are certain restrictions on the application of some materials, for instance, the power supply of electronic heating materials cannot work properly to supply heat under a low-temperature environment; the chemical reaction of the chemical energy heating materials is irreversible and can only be used as a disposable product. However, PCMs also rely on phase changes to release heat energy in low-temperature environments, and can be used to prepare heating and lightweight low-temperature protective materials.

Among the various PCMs, paraffin waxes are particularly attractive due to their high latent heat energy, nontoxicity, and chemical stability [21,22]. However, as solid-liquid PCMs, paraffin waxes are prone to leakage during the phase change process. To overcome this defect, many studies have been focused on the development of change phase microcapsules (microPCMs), which have a stable core-shell structure composed of an organic/inorganic shell and a PCM core [23,24,25]. In our previous work, we used microPCMs to prepare a low-temperature protective fabric [26]. The results showed that PCMs could play an active role in a low-temperature protection field. With the addition of microPCMs, the low-temperature resistance duration was prolonged, at an environment of −15 °C, −30 °C, −40 °C, and −60 °C, compared to pure fabric. To further improve the low-temperature protection performance, we also coated the outermost layer with silicone rubber [27]. Although silicone rubber has certain thermal insulation properties, it is obviously heavier than the commonly used textile material under the same volume conditions. Weight also affects the flexibility of the human’s activities, and it is difficult to sew silicone rubber. In order to solve the above problems, SiO_2_ aerogel with a light weight and excellent thermal insulation properties is used instead of silicone rubber. Therefore, in this paper, we designed and manufactured a multilayer low temperature protective composite fabric (MPF) based on active heating materials and passive insulating materials. The MPF had a three-layer structure, including a far-infrared fabric (the fabric layer), microPCMs (the function layer), and SiO_2_ aerogel (the thermal insulation layer). The function layer can offer heat energy and a thermal barrier against the effects of a low-temperature environmental. The insulation layer restrains the heat from the functional layer directly contacting the external cold environment, and the body heat and released heat dissipation can be suppressed. A combination of the two types of active and passive thermoregulating materials to design the MPF exhibit better low-temperature protection performance. N-octadecane (C18) was coated by melamine-urea-formaldehyde (MUF) and a oxygen-plasma-modified multiwalled carbon nanotube (CNT) hybrid shell to synthesize the microPCMs through in situ polymerization. Meanwhile, the far-infrared fabric, microPCMs, and aerogel were combined to prepare the MPF via coating technology. The morphology, phase-change behavior, low-temperature resistance, and thermal insulation performance of the MPF at −50 °C were tested and evaluated.

## 2. Experimental Section

### 2.1. Materials

C18 was the core, and Melamine (M), urea (U), and 37% formaldehyde solution (F) were the shell. The emulsifying agent selected sodium dodecyl sulfate (SDS). Triethanolamine and citric acid were used to adjust the pH. The diameters and lengths of the multiwalled CNTs were 10–50 nm and 10–30 μm, respectively. The mass fraction of the SiO_2_ aerogel slurry was 15%. Waterborne polyurethane (WPU, the content of the solid is 55%) was used as a binder agent. Polypropylene (PP) was spunbonded and nonwoven, with a mass per unit area of 40 g/m^2^. The polyester was spunlace far infrared nonwoven and polyester spunlace nonwoven, with a mass per unit area of 60 g/m^2^.

### 2.2. Synthesis of MicroPCMs

The MicroPCMs with an MUF/CNT hybrid shell was fabricated through in situ polymerization, and the method was the same as described in our previous studies [28]. This procedure included the preparation of the MUF/CNT prepolymer and oil-in-water (O/W) emulsion and the polymerization of the shell materials on the surface of the core droplets. In a typical synthesis process, the prepolymer solution was prepared by adding 3.81 g of melamine and 6.89 g of 37% formaldehyde into 70 mL of distilled water. Triethanolamine was used to adjust the pH of the mixture to 8.5, and the mixture was stirred at 70 °C to form a clear solution. Another 0.915 g of urea was added and stirred until it was dissolved. Meanwhile, 1.5 wt % CNTs was added and ultrasonically dispersed for 10 min. Then, 15 g of C18 was emulsified mechanically in a 10 wt % SDS solution at 40 °C for 30 min to generate a stable emulsion. The MUF/CNTs prepolymer was added dropwise into the emulsion with a stirring rate of 300 rpm, and the temperature was adjusted to 75 °C. After the prepolymer was completely added and the temperature reached 75 °C, the pH value of the system was adjusted to 5, with the citric acid solution, and the reaction lasted 3.5 h. Finally, by filtration and drying at room temperature, the microPCMs were obtained.

### 2.3. Preparation of the Thermal Insulation Layer

According to our previous work, increasing the thermal insulation layer is beneficial to low-temperature protection performance. In this paper, we selected the SiO_2_ aerogel slurry to prepare the thermal insulation layer. The slurry is formed by directly dispersing SiO_2_ aerogel into the water, which can be mixed with a binder and is conducive to the dispersion of SiO_2_. The preparation process of the thermal insulation layer was as follows: The SiO_2_ aerogel slurry and the WPU were mixed in a certain ratio and stirred to form a finishing solution. The spunbonded and nonwoven PP was fixed on the coating machine and the scraper height is adjusted to control a certain coating thickness. Then, the finishing solution was coated on the surface of the spunbonded and nonwoven PP through the coating machine. Finally, the SiO_2_ aerogel thermal insulation layer was acquired by drying at 50 °C.

The effect of the addition of aerogel slurry (0 wt %, 30 wt %, 50 wt %, 70 wt %, 80 wt %, coating thickness is 0.5 mm) and the coating thickness (0.5 mm, 0.75 mm, 1 mm, 1.5 mm, the amount of aerogel slurry is 70 wt %) on its morphology and thermal conductivity were discussed, and the process parameters for preparing the SiO_2_ aerogel thermal insulation layer were determined.

### 2.4. Preparation of the MPF

**Figure 1:**
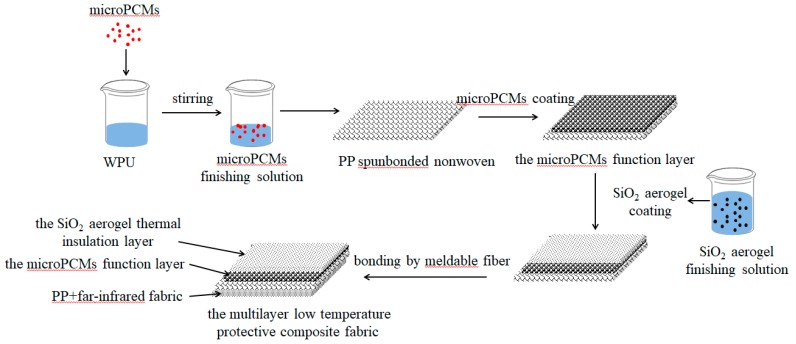
Preparation scheme for the multilayer low temperature protective composite fabric (MPF).

Figure 1 displays a simplified process diagram for the preparation of the MPF. Three key stages were included: (1) Preparation of the microPCM function layer, (2) preparation of the SiO_2_ aerogel thermal insulation layer, and (3) compound with far-infrared fabric.

The preparation parameters of microPCM function layer have been discussed in previous studies [29]. Firstly, 40 wt % microPCMs, 50 wt % WPU, and 10 wt % distilled water were mixed and stirred to form the finishing solution. The finishing solution was evenly coated with a 0.75 mm thickness on the surface of the spunbonded nonwoven PP at room temperature using the coating machine. Then, it was put into a dryer at 80 °C for 10 min to generate the microPCM function layer. Secondly, the surface of the micro PCM function layer was coated by the SiO_2_ aerogel using the preparation process described above. Finally, the far-infrared fabric was bonded to the other side of the spunbonded nonwoven PP, as mentioned above, by meldable fibers using an ironing machine to obtain the multilayer low temperature protective composite fabric.

### 2.5. Characterization

#### 2.5.1. Morphology

The surface morphology of the microPCMs was characterized using a Hitachi S-4800 scanning electron microscope (Hitachi, Tokyo, Japan) at an accelerating voltage of 10.0 kV. Tabletop SEM (TM 3030, Hitachi, Tokyo, Japan) was used to observe the surface and cross-section morphologies of the thermal insulation layer and the MPF. All the samples were coated with gold before observation. To better observe the cross-section morphology, the MPF was placed in a liquid nitrogen environment for a period of time and then cut.

#### 2.5.2. Particle Size Distribution

Image-Pro Plus analysis software was used to investigate the particle size distribution of the microPCMs, and at least 200 microPCMs were counted.

#### 2.5.3. Thermal Properties

A differential scanning calorimeter (DSC, Netzsch 204F1, Netzsch Group, Bavaria, Germany) was used to measure the phase change performances of the C18, the microPCMs, and the MPF. DSC measurements were carried out in the range of −20~60 °C, at a heating/cooling rate of 5 °C/min (C18, microPCMs) or 10 °C/min (the MPF) in a nitrogen atmosphere.

#### 2.5.4. Thermal Conductivity

The thermal conductivities of the thermal insulation layer and the MPF were measured with the Hot Disk Constant Analysers (TPS-2500S, Hot Disk, Uppsala, Sweden) at room temperature (the hot-disk sensor was a 7531 probe).

#### 2.5.5. Low-Temperature Resistance

**Figure 2:**
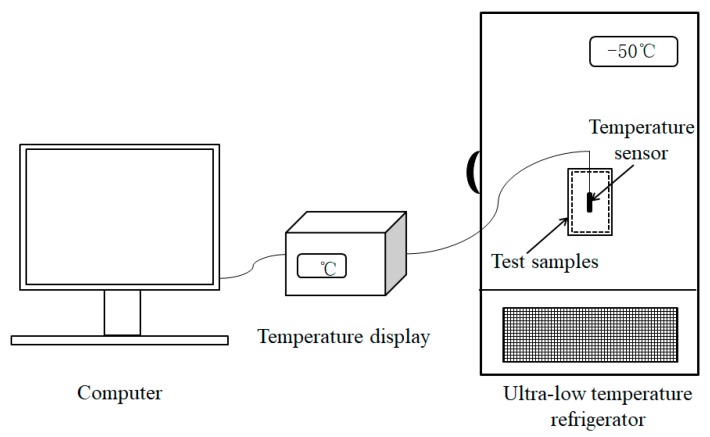
Low-temperature resistance test equipment.

Changes in the internal temperature of the MPF in a low-temperature environment were studied by an ultra-low temperature refrigerator (Zhongke Meiling Cryogenics Company Limited, Hefei, Anhui province, China) using the test equipment shown in Figure 2. A certain portion of the MPF was prepared in a pocket, and the temperature sensor was placed inside the pocket. The pocket was put in a 37 °C oven for a period of time and then removed and placed in an ultra-low temperature refrigerator for low-temperature resistance testing after reaching a heat balance. The temperature sensor recorded the internal temperature change of the MPF per second, and the experiment was terminated when the temperature dropped to 0 °C. The data were imported into the computer, and the temperature change curve of the MPF was plotted. The time for the internal temperature to change from its initial temperature to 0 °C was defined as the low-temperature resistance time of the MPF in this paper.

At present, the average annual temperature of the Antarctic continent is −40~−50 °C, according to relevant reports. The China Meteorological Administration has detected the average monthly temperature in the cold regions at around −30~−40 °C. For example, the average minimum temperature in Mohe (Heilongjiang province) in January 2019 was 32.5 °C, and the extreme minimum temperature of −58 °C occurred in 2017. In addition, the low temperature storage temperature in the biomedical field is usually 0~−60 °C. Considering the low temperature environment we used and the application temperature range of the MPF, the low temperature resistance performance under −50 °C was tested.

#### 2.5.6. Thermal Insulation Performance

**Figure 3:**
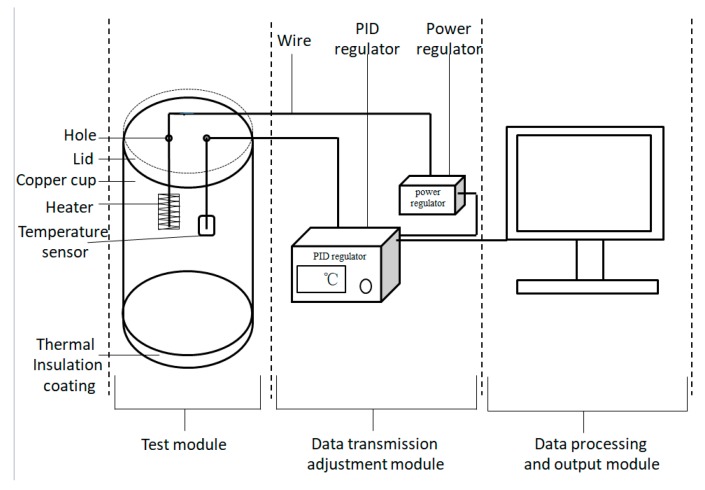
The test equipment for the thermal insulation performance in a low-temperature environment.

In order to access the thermal insulation performance of the MPF in a low-temperature environment, we designed the test equipment and the equipment structure as shown in Figure 3. The equipment mainly includes the test module, the data transmission adjustment module, and the data processing and output module (computer). The temperature sensor, heater and PID regulator were supplied by Beijing Tianyu Hengchuang Sensing Technology Co. Ltd. (Beijing, China). The power regulator was provided by Shanghai Hongshi Electronic Technology Co. Ltd. (Shanghai, China).

The main principle of this equipment has been described in the previous literature [27]. The specific operating steps for the test were as follows: The sample of a certain size was placed in an oven for thermal balance. Meanwhile, 500 mL of water was added into the test cup, and the target temperature (37 °C) was set by the PID regulator. After the sample reached thermal balance, and the water temperature in the test cup was stable at the target temperature, the sample was wrapped and fixed on the outside of the test cup, and the whole test module was placed in a low-temperature environment (−50 °C), while the computer was switched on to record data. After a while, the test module was removed from the low-temperature environment, and the experiment was finished. Finally, the data were processed by output module 3, and the power consumption needed to maintain the target water temperature during the test time was calculated. The thermal insulation performance was evaluated by the power consumption.

## 3. Results and Discussion

### 3.1. Morphology, Average Particle Size, and Thermal Properties of the microPCMs

**Figure 4:**
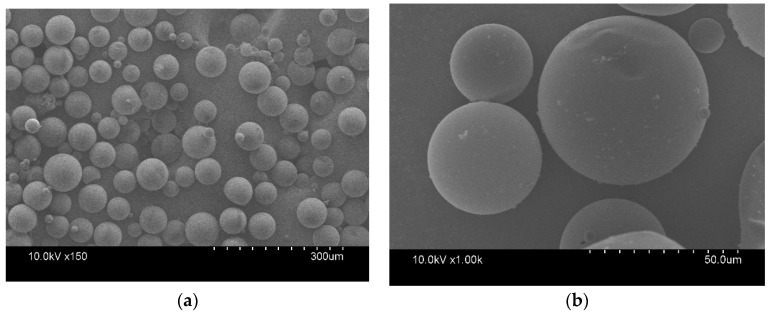
SEM images of the the phase change microcapsules (microPCMs): (**a**) ×150; (**b**) ×1000.

The surface morphology, average particle size, and phase change properties of the microPCMs are presented in Figure 4 and Table 1. It can be observed in Figure 4a that the majority of the microPCMs had a regular global shape without breaks or agglomeration. Based on the magnified SEM image (see Figure 4b), the surface of the microPCMs was relatively clean and smooth, indicating that the polymerization of the MUF/CNTs prepolymer had been well performed to synthesize a shell for the core. It is also notable that several small particles were coated on the surface of microPCMs. This phenomenon is related to the self-polycondensation of a small quantity of prepolymers [30,31].

Table 1 shows that the average particle size was 42.63 μm, and the melting (△*H*m) and crystallization (△*H*c) enthalpies of the microPCMs were 240.6 and 241.5 J/g, respectively. The encapsulated efficiency, η, still reached more than 79.57%. Thus, the microPCMs had excellent heat storage performance.

### 3.2. Effect of Aerogel Slurry Addition Amount on the Morphologies and Thermal Conductivity of the Thermal Insulation Layer

**Figure 5:**
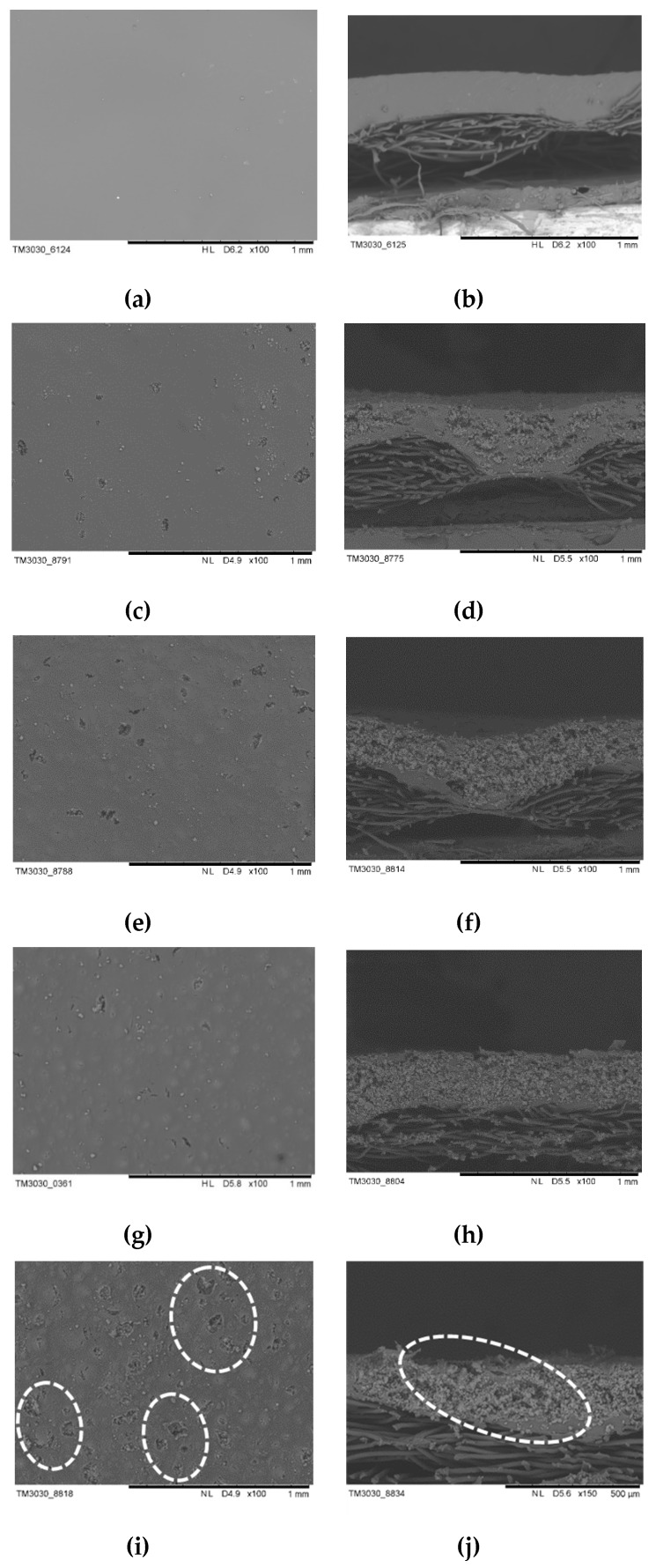
SEM images of the thermal insulation layer prepared at different SiO_2_ aerogel slurry addition amounts: (**a**,**b**) 0 wt %; (**c**,**d**) 30 wt %; (**e**,**f**) 50 wt %; (**g**,**h**) 70 wt %; (**i**,**j**) 80 wt %. the circles in (**i**,**j**) represent holes and cracks.

**Figure 6:**
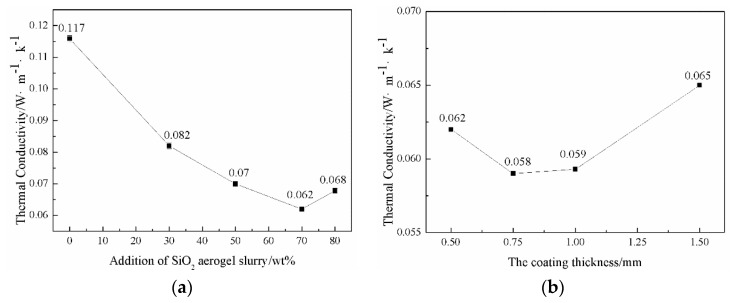
Thermal conductivity of the thermal insulation layer: (**a**) different SiO_2_ aerogel slurry addition amounts; (**b**) different coating thicknesses.

The morphologies and thermal conductivity of the thermal insulation layer, prepared at different SiO_2_ aerogel slurry addition amounts (the coating thickness was 0.5 mm), were characterized by SEM and hot disk; the results are presented in Figure 5 and Figure 6a. The surface and cross-section morphologies of the thermal insulation layer were obtained via SEM (Figure 5). It can be seen that the finishing liquid was not impregnated into the fabric and only adhered to the surface. The WPU could form a dense film on the fabric’s surface without the aerogel, and by increasing the aerogel slurry content, some small protrusions and particles on the surface of the film became more and more apparent. Meanwhile, the cross-section morphologies also showed that the particles increased significantly. The results indicate that the aerogel content in the thermal insulation layer gradually increased. When 80 wt % of the SiO_2_ aerogel slurry was added, the surface morphology of the thermal insulation layer presented some holes, and its cross-section morphology showed cracks. The results revealed that the content of the aerogel slurry in the finishing solution was too much and the WPU was not effective enough to form a complete film (which was not conducive to the adhesion of the aerogel on the surface of the fabric).

Thermal conductivity is one of the most crucial indexes to assess the performance of the thermal insulation layer. SiO_2_ aerogel plays an essential role in the thermal insulation layer. The thermal conductivity of the thermal insulation layer prepared at different SiO_2_ aerogel slurry addition amounts was summarized in Figure 6a. In a certain range, the thermal conductivity decreased with an increase in SiO_2_ aerogel content, which was related to the excellent thermal insulation properties of the aerogels. Low thermal conductivity is beneficial to low-temperature protection. However, at higher SiO_2_ aerogel addition amounts (80 wt %), a slightly increased thermal conductivity was acquired. The reason for this result is similar to that of the above morphologies. According to the above results, it can be seen that when the amount of aerogel slurry was at 70 wt %, the thermal insulation layer was complete and had a lower thermal conductivity.

### 3.3. Effect of Coating Thickness on the Morphologies and Thermal Conductivity of the Thermal Insulation Layer

**Figure 7:**
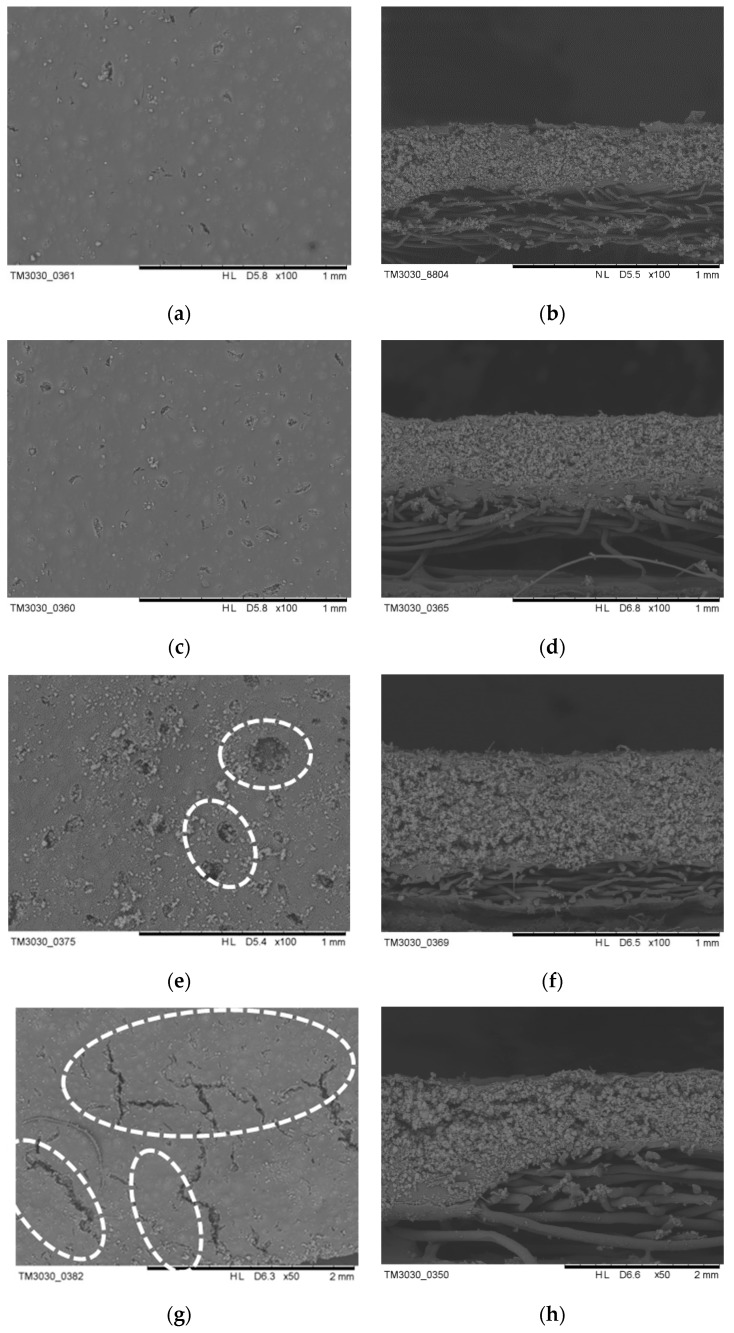
SEM images of the thermal insulation layer prepared at different coating thicknesses: (**a**,**b**) 0.5 mm; (**c**,**d**) 0.75 mm; (**e**,**f**) 1 mm, the circles in (**e**) indicate the holes; (**g**,**h**) 1.5 mm, the circles in (**g**) indicate the cracks.

Tuning the coating thickness of the thermal insulation layer is a critical approach to improve low-temperature protection performance. Detailed results about the morphologies and thermal conductivity of the thermal insulation layer prepared at different coating thicknesses (the amount of the aerogel slurry is 70 wt %) are presented in Figure 7 and Figure 6b. From the cross-section morphologies, it can be clearly seen that the insulation layer thickness and the aerogel attached to the fabric increase with an increase of the coating thickness. The surface morphologies also show that the surface integrity of the insulation layer becomes more and more visible as the coating thickness increases. The thicker the coating thickness, the longer the time required for drying, which causes the surface layer to naturally form a film, and the internal water vapor needs to pass through the surface layer, so the holes and cracks on the surface of the insulation layer are formed easily [32].

As shown in Figure 6b, the thermal conductivity of the insulation layer decreases gradually at first and then increases. Increasing the thickness can improve the thermal insulation performance and reduce the thermal conductivity but also easily leads to holes and cracks, which increase the heat transfer channel; in this way, the thermal conductivity increases. Therefore, the aerogel coating thickness is controlled at 0.75 mm.

### 3.4. Cross-Section Morphology and Thermal Properties of the MPF

**Figure 8:**
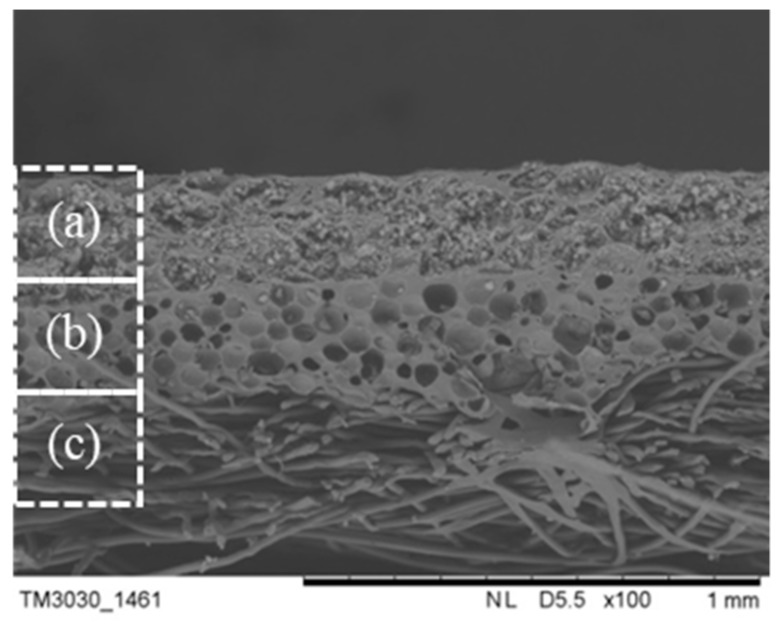
SEM image of the MPF: (**a**) the SiO_2_ aerogel thermal insulation layer; (**b**) the microPCM function layer; (**c**) PP+ the far-infrared fabric layer.

The MPF was prepared according to 2.4, and the preparation parameters of the thermal insulation layer were chosen based on the above results (the coating thickness was 0.75 mm, and the amount of the aerogel slurry was 70 wt %). The cross-sectional morphology of the MPF is displayed in Figure 8. It is obvious that in Figure 8, the MPF has a three layer structure including the fabric layer, the microPCM function layer, and the SiO_2_ aerogel thermal insulation layer. The composite effect between each layer is adequate, and there is no excessive penetration into each other, which could preserve the function of each layer. The fabric layer (PP+ far-infrared fabric) can be close to the body due to its softness and far-infrared function. Far-infrared fabrics can radiate far-infrared rays of a certain wavelength range to the human body. Far-infrared rays with a wavelength of 4~14 μm can penetrate through skin and gradually transfer energy into deep tissue via a resonance-absorption mechanism of organic and water molecules. In this way, skin blood microcirculation is promoted and a thermal response is produced. Moreover, far-infrared fabric can also reflect heat radiation back to the body and facilitate health care functions, such as activating the body, eliminating fatigue, improving immunity, etc. [33,34]. The microPCM function layer can be used as a heat source to release a certain amount of heat in a low-temperature environment. The outer layer utilizes the excellent thermal insulation properties of aerogel to retard the loss of internal heat.

**Figure 9:**
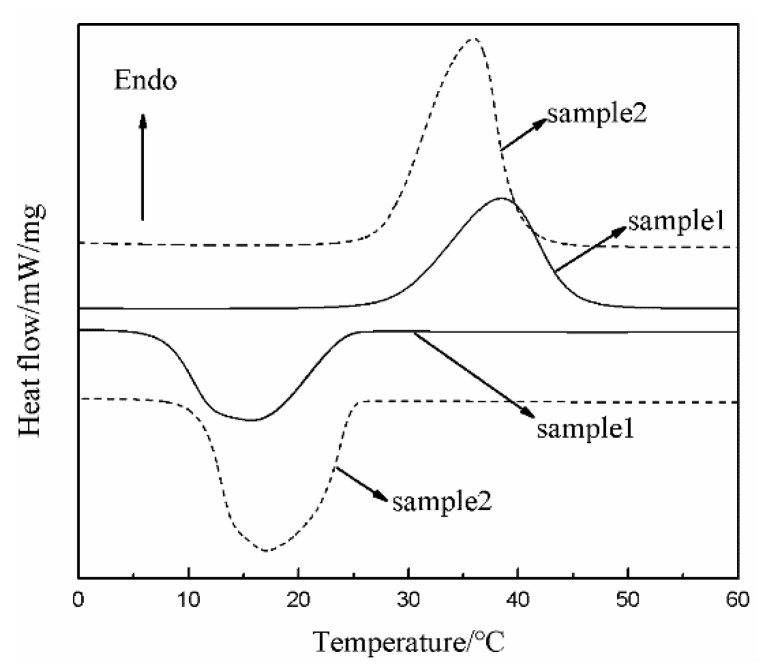
DSC curves of sample 1 and sample 2: Sample 1 was the MPF; sample 2 was the microPCM function layer with a coating thickness of 1.5 mm.

The DSC curve of sample 1 (the MPF) is presented in Figure 9. As shown, there is one endothermic peak and one exothermic peak during the heating/cooling process of the MPF. The Δ*H*m and Δ*H*c of the MPF were 96.1 and 96.2 J/g, respectively. Thus, the MPF can release heat energy in a low-temperature environment.

### 3.5. Low-Temperature Resistance of the MPF

**Figure 10:**
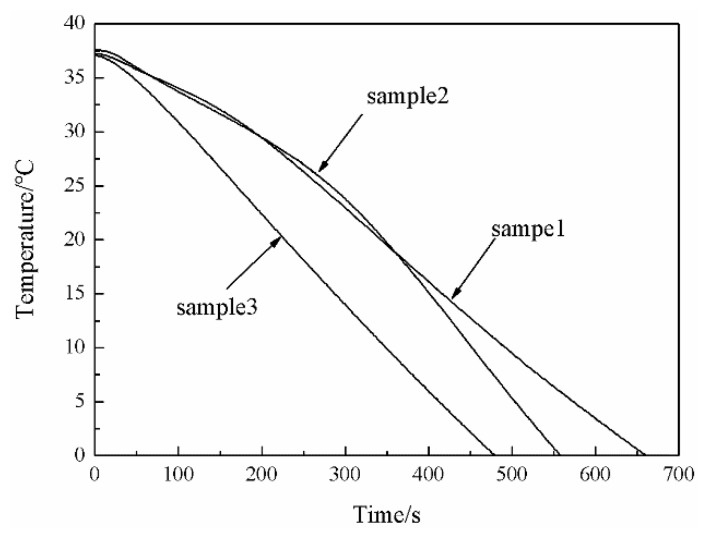
The low-temperature resistance: sample 1 was the MPF; sample 2 was the microPCM function layer with a coating thickness of 1.5 mm; sample 3 was the SiO_2_ aerogel thermal insulation layer with a coating thickness of 1.5 mm.

Figure 10 shows the low-temperature resistance curves of the MPF (sample 1), the microPCM function layer (sample 2), and the SiO_2_ aerogel thermal insulation layer (sample 3) with the same coating thickness (1.5 mm) as the MPF at −50 °C. The low-temperature resistance time of sample 1, sample 2, and sample 3 were 660, 558, and 487 s, respectively. Compared with sample 2 and sample 3, the low-temperature resistance time of sample 1 was extended by 102 and 173 s, close to 2 and 3 min, respectively. Moreover, by analyzing the three curves, we can clearly see that the slope of the temperature curve, which represents the different cooling rates, was different. In the initial stage of the curve, the cooling rate of sample 1 and sample 2 slowed down, and the trend persisted for a time, but sample 2′s cooling rate slowed down more obviously. This result is related to microPCMs releasing the latent heat to slow down the effects of temperature changes on the internal microclimate. It can be seen from the DSC curve (Figure 9) that the Δ*H*m and Δ*H*c of sample 2 were 138.1 and 139.5 J/g, respectively. The microPCMs containing C18 can store and release the latent heat. The greater the enthalpy value, the more microcapsules are contained. Under the condition of the same coating thickness, sample 2 contains more microPCMs and can release more latent heat at low temperature compared with sample 1, so the cooling rate slows down significantly at the initial stage. As the majority of the microPCMs have crystallized and the release heat gradually decreases, the cooling rate of sample 1 and sample 2 increased again, and the cooling rate of sample 2 was the fastest among the three curves. The reason for this result is that the released latent heat of sample 2 contacted the cold environment directly and the lost heat quickly. The MPF contains the fabric, microPCMs, and SiO_2_ aerogel, and the excellent thermal insulation performance of the outer layer prevents the heat from being released from the function layer that directly contacts and exchanges with the cold environment. The microPCMs in the function layer, as a kind of active heating material, could provide heat energy for the human body by their own phase change in a low-temperature environment. SiO_2_ aerogel, as a kind of passive insulating material, could slow down the heat loss from human body and heat energy from PCMs to the external environment due to its low thermal conductivity. A combination of the microPCM function layer (active heating materials) and the aerogel thermal insulation layer (passive insulating materials) was superior to the use of single materials, and the synergistic effect between the two could prolong the low-temperature resistance time and improve protection performance. The results confirmed our original design concept. Overall, the MPF was prepared based on active heating materials and passive insulating materials and met the protection requirements for low-temperature work over a short period of time.

### 3.6. Thermal Conductivity and Thermal Insulation Performance in the Low-Temperature Environment of the MPF

**Figure 11:**
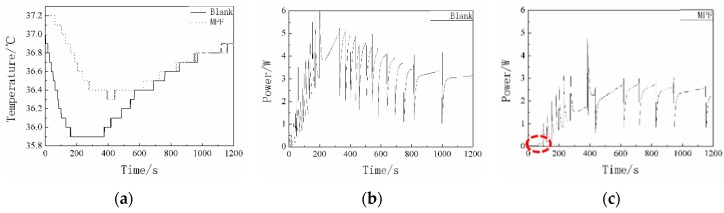
Thermal insulation performances in the low-temperature environment of the blank (The copper cup in the test module was not covered by the sample) and the MPF: (**a**) water temperature curve of the blank and the MPF; (**b**) the power consumption needed to maintain the water temperature of the blank; (**c**) the power consumption needed to maintain the water temperature of the MPF, the red circle in (**c**) highlights the MPF does not consume electrical power.

The thermal conductivity of the MPF was 0.0805 W/m K (as measured by Hot Disk Constant Analysers), which is smaller than the material we have produced [27]. To accurately evaluate the thermal insulation performance of the MPF in a low-temperature environment, the experiment was investigated by self-made equipment at −50 °C, and the results are shown in Figure 11 and Table 2. Figure 11 presents the water temperature variation and the power consumption curve of the blank (The copper cup in the test module was not covered by the sample) and the MPF. As seen in Figure 11a, the temperatures decrease at first and then rise within the error range during the test process due to the principle of the PID regulator. The specific reason for this phenomenon was explained in our previous paper [27]. In the initial stage of the test, it can also be seen that the water temperature of the MPF rose above 37 °C for nearly 200 s. Meanwhile, it is worth mentioning that the electrical power of the MPF was almost zero at the low temperature during the first 100 s (Figure 11c). This is related to the released latent heat via the phase transition of the C18.

The specific data of the thermal insulation properties are summarized in Table 2. From the data listed in Table 2, the power consumption for maintaining the target temperature of the blank and the MPF for 10 min was 1647 and 629 J, and for 20 min, it was 3472 and 1872 J. Compared with the blank, the power consumption of the MPF for 10 min decreased by 61.8%, and the consumption for 20 min decreased by 46.1%. The required power of the blank and the MPF were 3.3 and 2.5 W when the temperature was stable. The MPF clearly has excellent thermal insulation performance in a low-temperature environment.

In the MPF prepared by active heating materials and passive insulating materials, the aerogel was used to reduce heat loss while PCMs were used to release heat, and the thickness of the MPF was only about 1.4 mm. Therefore, based on the above analysis, the MPF we designed and manufactured will provide adequate protection effect for a short time while seldom affecting operation due to its light weight.

## 4. Conclusions

Microcapsules composed of a C18 core and MUF/CNT hybrid shell were synthesized using in situ polymerization. The microPCMs presented a regular global shape, and their average particle size was about 42.63 μm. A new-type multilayer low-temperature protective composite fabric was designed and manufactured based on active heating materials (the microPCMs we prepared) and passive insulating materials (SiO_2_ aerogel). An SiO_2_ aerogel thermal insulation layer with different morphologies and thermal conductivities were obtained by adjusting the amount of the aerogel slurry addition and the coating thickness. The thermal insulation layer had a complete morphology and a lower thermal conductivity when controlling the amount of the aerogel slurry. The aerogel’s coating thickness was 70 wt % and 0.75 mm, respectively. Moreover, the fabric layer, the microPCM function layer, and the SiO_2_ aerogel thermal insulation layer were combined to form the MPF. There was no excessive penetration between each element, so the function of each layer was preserved. The phase change enthalpy of the MPF for the cooling process was 96.2 J/g. The results also showed that the low-temperature resistance time of the MPF was 660 s at −50 °C. Compared with the same coating thickness of the microPCM function layer and the thermal insulation layer, the low-temperature resistance time of the MPF was extended close to 2 and 3 min, respectively. At −50 °C, the power consumption of the MPF (for maintaining the target temperature for 10 and 20 min) was 629 and 1872 J, respectively. The thermal insulation performance results also exhibited an excellent protective effect. In summary, by combining active heating materials with passive insulating materials, the MPF can better meet the demands of protection and operation under low-temperatures for a short time.

## Figures and Tables

**Table 1:** The Phase Change Properties of C18 and MicroPCMs.

Samples	Average Particle Size (μm)	*T*_m_ (°C)	Δ*H*_m_ (J/g)	*T*_c_ (°C)	Δ*H*_c_ (J/g)	η (%)
C18		27.2	307.1	25.5	298.8	
microPCMs	42.63	26.8	240.6	25.7	241.5	79.57

**Table 2:** Thermal insulation results.

Sample	Power at Stable Temperature/W	The Power Consumption/J	
		10min	20min
Blank	3.3	1647	3472
MPF	2.5	629	1872
